# Control of actin dynamics during cell motility

**DOI:** 10.12688/f1000research.18669.1

**Published:** 2019-11-25

**Authors:** Simona Buracco, Sophie Claydon, Robert Insall

**Affiliations:** 1 Institute of Cancer Sciences, University of Glasgow, Bearsden, G61 1BD, UK

**Keywords:** Cytoskeleton, actin, arp2/3 complex, formin, vesicle, traffic, cell motility, migration, chemotaxis

## Abstract

Actin polymerization is essential for cells to migrate, as well as for various cell biological processes such as cytokinesis and vesicle traffic. This brief review describes the mechanisms underlying its different roles and recent advances in our understanding. Actin usually requires “nuclei”—preformed actin filaments—to start polymerizing, but, once initiated, polymerization continues constitutively. The field therefore has a strong focus on nucleators, in particular the Arp2/3 complex and formins. These have different functions, are controlled by contrasting mechanisms, and generate alternate geometries of actin networks. The Arp2/3 complex functions only when activated by nucleation-promoting factors such as WASP, Scar/WAVE, WASH, and WHAMM and when binding to a pre-existing filament. Formins can be individually active but are usually autoinhibited. Each is controlled by different mechanisms and is involved in different biological roles. We also describe the processes leading to actin disassembly and their regulation and conclude with four questions whose answers are important for understanding actin dynamics but are currently unanswered.

## Introduction

Actin, one of the two core components of the eukaryotic cytoskeleton, together with tubulin, is a small globular protein that is one of the most abundant proteins in many cells. Both polymerize into filaments that are absolutely required for several aspects of normal cell physiology.

Actin is best known in two structures that are connected with movement. Muscle is a stable, almost crystalline array of parallel α-actin and myosin filaments. Upon activation, the myosin molecules pull on the actin filaments, converting energy in the form of ATP to contraction. The actin of non-muscle cells, made of two slightly different isoforms (β- and γ-) is much more dynamic and complex, comprising both the fairly stable cortex and a range of highly dynamic protrusions at the edges of cells, in which actin filaments have lifetimes of tens of seconds. It remains unclear why nature uses near-identical molecules for structures with such contrasting lifetimes. A more recently appreciated feature of non-muscle actin is its role in vesicle dynamics. Vesicles of multiple classes require actin to bud and sort correctly. This review describes past and recent progress in the physiological roles of actin polymerization and how it is controlled.

## Control of actin nucleation

**Figure 1.**
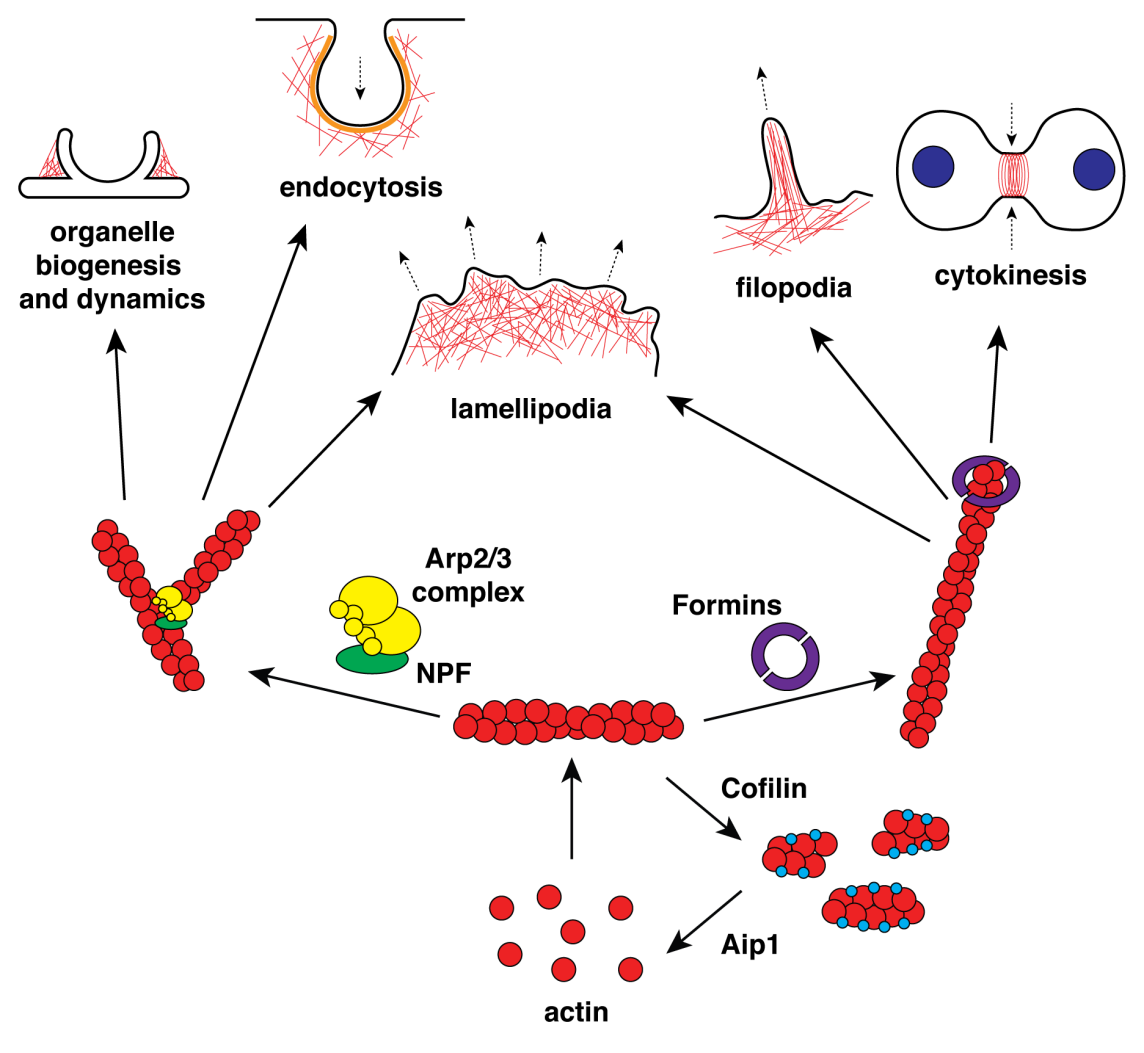
Schematic diagram showing key pathways controlling actin assembly. Aip1, actin-interacting protein 1; Arp2/3, actin-related protein 2/3; NPF, nucleation-promoting factor.

The generation of new actin filaments requires the assembly of two or three monomers that act as templates for polymerization and filament growth. This process, known as “nucleation”, is kinetically unfavorable: actin dimers are unstable, and most actin monomers are sequestered by actin monomer-binding proteins such as profilin and thymosin β4, which prevent spontaneous nucleation of new filaments. Thus, nucleation is a rate-limiting step in the assembly of new actin filaments ^1^. Cells overcome this kinetic barrier by employing “actin nucleation factors” that catalyze the *de novo* formation of actin filaments. Of the several actin nucleation factors that have been described so far, the actin-related protein 2/3 (Arp2/3) complex and formins ( Figure 1) are the best studied and appear the most physiologically important ^2^.

## Arp2/3 complex

The Arp2/3 complex was the first major actin nucleator to be identified ^3^. This complex is a large (~220 kDa) stable assembly of seven subunits all highly conserved in eukaryotes: Arp2, Arp3, and five other supporting subunits named ArpC1 (p40), ArpC2 (p34), ArpC3 (p21), ArpC4 (p20), and ArpC5 (p16). *In vitro* studies demonstrated that, rather than nucleating *de novo* actin filaments, the Arp2/3 complex promotes the assembly of a new filament from the side of a pre-existing one at a 70° Y-branched angle ^4^. Both Arp2 and Arp3 structures resemble the actin fold, and the two subunits together mimic an actin dimer from which nucleation can take place. The nascent filament is capped at its slow-growing pointed end by the complex, linking it firmly to the older filament, but is free to elongate in the fast-growing barbed direction ^5^.

The Arp2/3 complex alone is an inefficient nucleator: its Arp2 and Arp3 subunits are too far apart to nucleate a new filament, and its binding to the side of an actin filament is kinetically unfavorable. However, its ability to promote actin polymerization is strongly increased by the engagement of nucleation-promoting factors (NPFs). The main NPFs of the Arp2/3 complex are members of the Wiskott-Aldrich syndrome protein (WASP) family, which includes WASP and N-WASP, Scar/WAVE, WASH, WHAMM, and JMY (the acronyms Scar, WAVE, WASH, WHAMM, and JMY derive from “suppressor of cAMP receptor mutants”, “WASP family Verprolin-homologous protein”, “WASP & Scar homologue”, “WASP homolog associated with actin, membranes, and microtubules”, and “junction-mediating regulatory protein”, respectively). These NPFs contain a conserved C-terminal VCA domain, which consists of one or more verprolin homology domains (V) that bind actin monomers, and a central amphipathic linker (C) and an acidic region (A) that together bind the Arp2/3 complex ^6^; it is believed that WASP proteins recruit actin monomers through their V region and bind Arp2/3 complex through the C and A portions ^7^.

It is traditional to consider actin and the Arp2/3 complex as primarily motility proteins, but it is striking how vesicle-centered the NPF family as a whole is: Scar/WAVE is the key activator of pseudopods and lamellipods, but WASH, WHAMM, and JMY are purely vesicular, and WASP/N-WASP are now thought to have mainly endocytic roles.

Recent work has progressed our understanding of the role of NPFs in the activation of the Arp2/3 complex. Chemical cross-linking of cysteines engineered in Arp2 and Arp3 has been used to hold yeast Arp2/3 complex in a short-pitch conformation ^8^. When cross-linked in this conformation, the complex is hyperactive and bypasses the need for WASP in activation. Moreover, its activity is even higher than WASP-activated Arp2/3 complex, indicating that the cross-link stabilizes an activated state of the complex. This confirms that WASP’s main activating function consists in its ability to move Arp2 and Arp3 closer in a short-pitch active conformation. More recently, these results have been extended using fluorescence spectroscopy and EM to determine the conformational changes induced by NPF binding to the Arp2/3 complex ^9^. As earlier suspected, VCA binding causes a large conformational change that moves Arp2 closer to Arp3. Moreover, it also favors the complex binding to the side of an actin filament, leading to further rearrangements that are required for nucleation of a new actin filament.

WASP-activated Arp2/3 complex can nucleate new actin only when templated by an existing filament. Thus, the generation of branched actin networks has been thought to require priming by nucleators other than the Arp2/3 complex. However, a newly identified class of NPFs, named WISH/DIP/SPIN90 (WDS) proteins, may activate the Arp2/3 complex without the presence of a preformed filament and form linear filaments ^10^. Recently, these linear actin filaments generated by WDS-bound Arp2/3 complex have been found to act as initial seed filaments, upon which WASP-activated Arp2/3 complex can bind and catalyze the formation of branched actin ^11^.

The Arp2/3 complex is unique among nucleators because it catalyzes the formation of branched filaments, instead of separate linear ones. The resulting “dendritic” network is responsible for the formation of several actin structures, including lamellipodia ^12^ and the actin patches at the sites of clathrin-mediated endocytosis ^13^. Actin dynamics driven by WASH have not often been consistently visualized, but, in mutants that cause excess activity, lamellipod-like actin may be seen streaming off post-lysosomes ^14^. In addition to these well-known functions, a link between Arp2/3 complex-mediated actin assembly and mammalian autophagy has been suggested. An actin branched network generated by the Arp2/3 complex is essential for autophagosomal membrane shaping ^15^, and two NPFs, WHAMM ^16^ and JMY ^17^, colocalize with autophagosome markers and play a role in autophagosome biogenesis. In particular, LC3 and stress-responsive activator of p300 (STRAP) were recently identified as regulators of JMY activity during autophagosome formation: LC3 recruits JMY to the membrane and stimulates its actin nucleation activity, while STRAP inhibits JMY and antagonizes its activation mediated by LC3 ^18, 19^.

Thus, Arp2/3 complex-dependent actin nucleation is involved in the organization of several organelles, including endosomes, lysosomes, and autophagosomes. This list has been recently extended to include mitochondria: a crosstalk between branched actin and microtubules has been proposed to control mitochondria distribution ^20^. This regulation appears to be particularly important, as mitochondrial dynamics affect most cellular processes, including cell migration ^21^ and invasion ^22^.

The Arp2/3 complex activity has also been recently shown to be involved in the generation of a dynamic perinuclear actin network that allows cells to deform their nucleus and go through narrow pores. This mechanism is used by dendritic cells to move from peripheral tissues to lymph nodes for antigen presentation ^23^, but it could also be used by metastatic cells to migrate through dense tissues.

One key question in recent years surrounds the essential nature—or otherwise—of Arp2/3 complex in migration. Two near-simultaneous papers, using similar cells, came to opposite conclusions. The first found that the Arp2/3 complex was absolutely essential for directed motion ^24^; the second found it to be dispensable ^25^. We consider real biology to be more nuanced. The paper by Wu *et al*. clearly shows that directed motion is possible under specific circumstances. Similar results—from dendritic cells lacking Scar/WAVE and therefore unable to assemble Arp2/3 complex at the leading edge ^26^—show that directed migration is possible but hugely different from normal Arp2/3 complex-driven migration. We conclude that actin-driven migration can be driven by multiple parallel mechanisms. The Arp2/3 complex dominates the process in normal cells, but in its absence migration is possible, if too inefficient for most physiology. Curiously, in *Dictyostelium* cells, loss of Scar/WAVE is less damaging because WASP can be repurposed and take over ^27^. The implications for upstream signaling are complex and not understood, but this work does show that Arp2/3 complex assembly is fundamentally similar between different nucleators.

## Formins

Another key player, with a contrasting role in the control of actin assembly, is the formin family of proteins (more thoroughly reviewed in ^28^). Formins were first described in 1990, following a screen to identify genes involved in mouse limb deformities ^29^. Subsequent structural studies of these proteins identified homology domains and the link to the actin cytoskeleton was established ^30^. Initial studies in yeast showed that formin proteins were essential for the formation of actin bundles that conferred polarity upon the cells and that this was regulated by the Rho GTPase network via the formin homology domains ^31^. Formins were then shown to nucleate actin filaments by *in vitro* experiments with purified proteins, with the FH2 (formin homology 2) domain playing an essential role via its ability to bind the barbed end of actin filaments ^32^. However, unlike other proteins which bind the barbed end and halt elongation, formins have been described as “leaky cappers”, referring to their ability to cap the filament while still allowing actin monomers to join and elongation to continue ^33^. After nucleation, the formin FH2 domain encircles the tip of the barbed end and allows filament elongation. All formins slow elongation owing to an equilibrium between conformations that block or allow subunit addition ^34, 35^. This inhibition varies from about 5 to 95% depending on the formin. Delivery of actin-profilin from binding sites on FH1 domains to the barbed end can increase the rate of elongation beyond the rate of a bare filament end depending on the formin in question, its conformations, and the concentrations of profilin and other proteins ^35^. These differences in elongation rates suggest a “gating model” in which a more open conformation of the FH2 domain favors monomer addition and therefore elongation, and a closed conformation inhibits the process ^35^. Differing models have proposed how this inhibition and the open and closed conformations occur. Recently, simulation data focused on analysis of the gating model have suggested that the FH2 domain of formins can cause this effect by both steric blocking of actin subunits at the barbed end of the filament and changing the helical twist of the filament which interferes with availability of the binding site ^36^.

After nucleation, the FH2 domains of the formins remain attached to the barbed end, encircling the growing filament, unlike the Arp2/3 complex, which is drawn backwards into the cell with bulk actin. The key to understanding formins is that their roles tend to be narrow: individual formins are used for specific processes, unlike the broad roles of Arp2/3 complex (though the effects of deleting them may be additive ^37^). Formins play a critical role in filopodia formation. This result is reinforced by studies of the homologous dDia2 in *Dictyostelium*, which not only localizes to the tips of filopodia and regulates their formation but also contributes to polymerization by competing with capping proteins at the barbed end of the filaments ^38^. dDia2 mutants have a much-reduced rate of filopod production. A recent study has shown that formins and capping proteins can bind the barbed end of actin filaments simultaneously, leading to a weakened state of binding of both ^39^. This adds complexity to their interaction and subsequent effect on elongation, with the possibility that capping proteins can also compete with formins for binding and thereby inhibit elongation.

Force can influence the rate of actin elongation by formins. After speculation that a pulling force applied to FH2 rings at the barbed end of actin filaments might increase the rate of elongation ^40^, studies using buffer flow to exert force on actin filaments showed formins respond differently. Force increases the rate of elongation by mDia1 ^41^ but decreases the rate of elongation by yeast Bni1 in the absence of profilin and increases elongation in the presence of profilin ^42^. Force exerted by magnetic tweezers also increases the rate of elongation by mDia2, especially if the FH2 ring is allowed to rotate around the bound filament ^43^.

Formins can collaborate with the Arp2/3 complex. As well as their role in linear filament and filopodia formation, they are also important in the branched actin of lamellipodia. mDia2 depletion in the cell inhibits lamellipodia formation, and it was speculated that this was due to the formins protecting the growing filaments from capping proteins that would halt their protrusion ^44^. More recently, this idea has been explored further in another subfamily, the FMNL formins. In B16F1 melanoma cells, FMNL2 localizes at the lamellipodia edge as well as at filopodial tips. Biochemical experiments revealed that FMNL2 more efficiently participated in the elongation of filaments rather than the nucleation and that in fact it could elongate filaments nucleated by the Arp2/3 complex, explaining its localization. The disruption of FMNL2 and FMNL3 in cells led to an overall reduction in lamellipodial migration efficiency ^45^. Another study using B16F1 cells even more recently showed that when FMNL2 (along with FMNL3) activity is abolished, lamellipodial dynamics and appearance are quite different. Lamellipodia become narrower, with less intense actin filament networks at the leading edge. This disruption of FMNL2 also decreases the force generated by B16F1 cells and impacts their ability to push whilst migrating ^46^. Taken together, these studies clearly demonstrate that the reality of actin filament assembly is not as simple as “formins nucleate filopodial actin filaments and the Arp2/3 complex nucleates lamellipodial filaments” and that they can cooperate. It is also important to remember that the Arp2/3 complex is an amplifier of existing actin, rather than a creator *ab initio*. Arp2/3 complex requires pre-existing (and preferably new) actin filaments to grow; formins probably provide the template.

Interestingly, it appears that formins’ role in filopodia is not only to nucleate/elongate filaments but also to play a part in adhesions. A recent study sought to investigate the mechanisms by which filopodia respond to force and how this affects their adhesion to the extracellular matrix ^47^. The authors examined the growth of filopodia that were attached to fibronectin-coated beads which were under a constant pulling force and found that the adhesion to these beads and the growth caused by the force was dependent on myosin IIA and formin mDia2. When these proteins were inhibited, both the growth and the force exerted by the filopodia were significantly diminished ^47^. This study provides a crucial insight into a new role for formin in actin dynamics *in vivo*, where cells are constantly responding to forces from both within the cell and their environment.

## Spatial and temporal coordination of the actin machinery

As discussed in previous sections, cells simultaneously assemble, maintain, and disassemble actin filaments to generate a wide array of actin architectures. This complexity requires precise spatial and temporal coordination at different levels and the ability to rapidly and efficiently answer to intracellular and extracellular signals. Despite continuing progress in understanding how actin structures are assembled, less is known about how the actin nucleating systems are integrated. However, several papers in recent years have addressed this question.

Arp2/3 complex and formins nucleate different actin filaments from a common limited pool of actin monomers. High concentrations of G-actin are maintained in the cell by the cooperation of actin monomer-binding proteins such as profilin and capping proteins ^1^. In addition, profilin distributes actin monomers between filaments nucleated by Arp2/3 complex and formins in both fission yeast ^48, 49^ and animal fibroblasts ^48, 49^. When the Arp2/3 complex is present in excess, profilin can inhibit its nucleating activity by disrupting the association between WASP proteins and actin. By doing so, it favors formin-mediated actin nucleation and maintains the homeostasis between the two main actin assembly machineries ^48, 49^. More generally, competition for resources is clearly fundamental to actin function: WASP, Scar/WAVE, and formins all compete for a limited pool of actin monomers, and imbalances caused by the loss of one can deregulate the others ^50, 51^.

Spatial differentiation of the many actin-binding proteins is crucial for subcellular compartmentalization. Inositol lipids are essential, though their roles are complex and subject to misinterpretation. Several studies of actin waves—structures that may represent frustrated phagosomes or may be fundamental mechanisms underpinning normal protrusion dynamics—address the interplay between lipid composition of the plasma membrane and actin dynamics ^52– 56^. During frustrated phagocytosis in mammalian macrophages, the plasma membrane enclosed by F-actin waves shows polarized distribution of both actin and lipids. In this region of plasma membrane, cortical actin is absent and the lipid composition is altered, with depletion of phosphatidylinositol 4,5-bisphosphate (PI[4,5]P _2_) and enrichment of PI(3,4)P _2_^57^. However, altering actin polymerization by the use of an N-WASP inhibitor causes a rapid loss of the lipid polarized distribution. This implies a positive feedback from the actin network to lipid-modifying enzymes. At the same time, the lipid composition of the plasma membrane can control and modify actin assembly.

The interplay between the plasma membrane and actin network is dependent not only on its phosphoinositide composition but also on its curvature. Indeed, high membrane curvature has been shown to increase PI(3,4)P _2_ dephosphorylation, starting a cascade of reactions that alter the lipid composition of the membrane and activate the actin machinery at specific cellular locations ^58^.

The upstream control of the NPFs has also been a mystery. The small GTPase Rac is clearly essential for the activation of the Scar/WAVE ^59^. However, its mechanism of action has been opaque. One important reason for this is the large five-subunit complex in which it is found; the intact complex seems excessively large (around 500 kDa, depending on the source) and complicated. Rac binds to one subunit (confusingly, the same subunit is named Sra1, CYFIP, or PIR121 by different authors). Initially this interaction was described as a simple interaction with an N-terminal domain ^60^, but this is complicated by the discovery of a possible second binding site ^61^. Genetic analysis in melanoma cells and *Dictyostelium* show the N-terminal site is essential for Scar/WAVE to function, while the second is important for its efficiency ^62^. A second protein, CYRI, shares a similar N-terminal domain but appears to act as a competitive inhibitor that limits actin polymerization ^63^. WASH regulation is similarly complex and similarly poorly understood. Like Scar/WAVE, WASH is found in a large five-membered complex, which, very unusually, is assembled using a dedicated chaperone ^64^. Reports that WASH is directly regulated by small GTPases are at best controversial, but it is clearly regulated by covalent modifications like ubiquitination ^65^.

## Actin disassembly

Whilst the assembly of actin filaments is vigorously studied to explain how cells achieve motility, it is also necessary to understand how the filaments disassemble. However, much less is known about this. The pool of actin monomers must be maintained to allow rapid polymerization of new or existing filaments ^66^. After nucleation of an actin filament by the Arp2/3 complex or formins, actin filaments can naturally assemble new monomers at the barbed end and disassemble monomers from the pointed end. This is a process known as actin treadmilling ^67^, which depends on the hydrolysis of ATP bound by the assembled actin monomers and dissociation of the γ-phosphate from the terminal subunits ^68^.

Although actin treadmilling explains the natural turnover of filaments *in vitro*, it cannot account for the rapid and varied extents of actin disassembly in the cell. ADF/cofilin participates in active severing of actin filaments ^69^. The mechanism by which cofilin regulates actin turnover has been studied in detail, and it is known that the binding of cofilin to the filament induces a helical twist ^70^. This causes an increase in flexibility of the areas of the filament bound by cofilin, leaving the unbound areas stiff ^71^. Severing can then occur between these areas, as the difference between the bound and unbound areas puts stress on the filament ^72^. Although cofilin has a higher affinity for ADP actin monomers than for ADP-Pi actin monomers or ATP actin monomers in the filament ^73^, cofilin can bind the ADP-Pi actin monomers and promote the release of the γ-phosphate ^74^.

Subsequent work showed that cofilin works in combination with actin-interacting protein 1 (Aip1), which drives disassembly through a different mechanism ^75^. The mechanism by which this occurs has been explored in different ways. One approach investigated how these proteins work together by examining the effect they have on different actin filament architectures ^76^. Using protein micropatterning of an NPF in combination with Arp2/3 complex and actin, the authors were able to reconstruct three different kinds of actin networks: Arp2/3 complex branched networks, polarized actin cables, and non-polarized actin filaments. ADF/cofilin and Aip1 acted differently on these structures ^76^. While ADF/cofilin alone was sufficient to disassemble the Arp2/3 complex branched networks, the addition of Aip1 was needed to act on the others. Another interesting observation was that the timing of the addition of these proteins also changed the outcome. When ADF/cofilin was added before the Aip1, the disassembly when the Aip1 was eventually added was much greater than if both proteins were added simultaneously. The authors propose that ADF/cofilin actin binding limits the rate at which Aip1 can act and that the shape change of a filament when ADF/cofilin binds acts as a scaffold for Aip1 activity. An interesting remaining study is to explore further the action of the Aip1 on disassembly after it is recruited to ADF/cofilin-bound actin ^76^.

An alternative line of research involved manipulating the concentration of cofilin. The ratio of cofilin to actin drastically changes the effect on the filament, with lower cofilin densities favoring severing and higher densities favoring stabilization ^77^. However, recent work found actin filaments in thymus extract can disassemble quickly in spite of high cofilin concentrations. The authors used imaging of single actin filaments and determined that Aip1 was responsible for this, allowing the filaments to quickly switch from a stable state to rapid disassembly ^78^. This highlights an important mechanism by which cells can switch their actin networks from stable to unstable to allow dynamic changes in architecture. It is also now known that Aip1 binds to the side of actin filaments, possibly competing with cofilin for binding to create unbound areas that promote severing ^79^. Further work using advanced single molecule imaging has highlighted the importance of coronin1B for this process, as it binds to actin filaments leading to the recruitment of cofilin and saturation of the filament before Aip1 promotes severing amongst the high concentration of cofilin ^80^. The authors have since further explored this process and determined that different isoforms of tropomyosin which bind actin protect and stabilize the filaments from this process to different extents, identifying another layer of complexity to the control of disassembly ^81^.

## Unanswered questions

Despite the large volume of work briefly summarized in this review, we still lack a coherent picture; many individual factors that control actin are known, but it is still not possible to describe the global mechanisms that make actin appear in some places and not others. Obvious unanswered questions include:

(1) Why are large complexes needed? There seems to be no logic for the Arp2/3 complex to have seven members, given our current understanding of its function. Arp2 and Arp3 have obvious roles, but it seems hugely complex to use five other subunits to connect them to an established filament. One answer may come from different subunits that modulate the complex’s behavior ^82^. However, simpler organisms like yeast and *Dictyostelium* have single forms of each subunit, so this seems a refinement for multicellular organisms rather than a fundamental feature. The Scar/WAVE and WASH complexes have only five members each but are huge (around 450 kDa) despite described functions that are achieved efficiently with single, smaller proteins. Is the large size of these complexes somehow physically important to their function? Or do they have large numbers of interactions that remain unknown?

(2) How do the multiple small GTPases of the Rho, Rac, and Cdc42 families collectively regulate actin? Actin’s polymerization occurs in an extremely complex and localized architecture, but most experiments suggest that activated GTPases are rather diffusely localized. How can diffuse signals specify finely regulated structures? One possible answer is positive feedback, in which actin polymerization is the proximal stimulus for more actin polymerization. This has been generally accepted to be important since the seminal work of Vicker (for example ^83^); positive feedback loops can produce unpredictable and complex patterns from simple inputs.

(3) However, if positive feedback is the key, how does it work? Most authors have looked at feedback at the level of signals such as small GTPases, but that cannot answer the previous question. There is presumably a mechanism by which new actin causes the initiation of yet more actin filaments, for example by the Arp2/3 complex showing a preference for free barbed ends ^84^. Full understanding of this process is fundamentally important for the field.

(4) Finally, what are the roles for IQGAP ^85^, VASP ^86^, and CAP ^87^? A range of data show each protein is fundamentally important, but each paper shows glimpses of their roles without offering a compelling mechanism. Our ultimate goal should be a complete, informative mathematical model showing how actin works during migration, as we can achieve for signaling during chemotaxis ^88^. To construct such a model, we need a more complete understanding of all of the key components of actin signaling and their interactions.
